# Pathophysiology of Chemotherapy-Induced Peripheral Neuropathy

**DOI:** 10.3389/fnmol.2017.00174

**Published:** 2017-05-31

**Authors:** Hana Starobova, Irina Vetter

**Affiliations:** ^1^Centre for Pain Research, Institute for Molecular Bioscience, University of QueenslandSt Lucia, QLD, Australia; ^2^School of Pharmacy, University of QueenslandSt Lucia, QLD, Australia

**Keywords:** cancer, chemotherapy, neuropathy, pain, vincristine, paclitaxel, oxaliplatin, cisplatin

## Abstract

Chemotherapy-induced neuropathy is a common, dose-dependent adverse effect of several antineoplastics. It can lead to detrimental dose reductions and discontinuation of treatment, and severely affects the quality of life of cancer survivors. Clinically, chemotherapy-induced peripheral neuropathy presents as deficits in sensory, motor, and autonomic function which develop in a glove and stocking distribution due to preferential effects on longer axons. The pathophysiological processes are multi-factorial and involve oxidative stress, apoptotic mechanisms, altered calcium homeostasis, axon degeneration and membrane remodeling as well as immune processes and neuroinflammation. This review focusses on the commonly used antineoplastic substances oxaliplatin, cisplatin, vincristine, docetaxel, and paclitaxel which interfere with the cancer cell cycle—leading to cell death and tumor degradation—and cause severe acute and chronic peripheral neuropathies. We discuss drug mechanism of action and pharmacokinetic disposition relevant to the development of peripheral neuropathy, the epidemiology and clinical presentation of chemotherapy-induced neuropathy, emerging insight into genetic susceptibilities as well as current understanding of the pathophysiology and treatment approaches.

## Introduction

Chemotherapy-induced peripheral neuropathy (CIPN) is an adverse effect of many chemotherapeutic agents and a major cause of ongoing pain in cancer survivors (Farguhar-Smith and Brown, 2016). There are six main substance groups that cause damage to peripheral sensory and motor neurons, resulting in development of CIPN: the platinum-based antineoplastics (particularly oxaliplatin and cisplatin), the vinca alkaloids (particularly vincristine and vinblastine), the epothilones (ixabepilone), the taxanes (paclitaxel, docetaxel), the proteasome inhibitors (bortezomid) and immunomodulatory drugs (thalidomide). The high success rate of these drugs in cancer treatment has led to a steady increase in the survival rates of patients. Consequently, the number of cancer survivors suffering from neuropathic pain conditions is rising as well. Overall, approximately 68% of patients receiving chemotherapy develop CIPN within the first month of treatment (Seretny et al., 2014), the development of which is related to both single as well as cumulative drug doses. Additionally, various conditions like pre-existing nerve damage, for example in diabetic patients, can be linked to an increased risk of developing CIPN.

Although the mechanisms of action and molecular target(s) of these chemotherapeutic agents are diverse and include both DNA and microtubular targets to arrest cell division and induce cell death, the pathobiology of chemotherapy-induced neuropathy, irrespective of the causative agent, shares some important similarities. Notably, CIPN is characterized by predominantly sensory axonal peripheral neuropathy (Quasthoff and Hartung, 2002) that typically develops in a “stocking and glove” type distribution, with longer axons affected first (Han and Smith, 2013). The histopathological changes associated with CIPN commonly involve large myelinated fibers, although bortezomib-induced neuropathy also involves small fibers (Cata et al., 2007; Wilkes, 2007; Gutierrez-Gutierrez et al., 2010). These changes to the morphological and molecular physiology of peripheral nerves result in the development of sensory and motoric symptoms such as hypersensitivity to mechanical stimuli or distal weakness due to mechanisms which are not entirely understood.

The symptoms of CIPN can be severe; management with common analgesic approaches are often unsatisfactory; and despite increasing insights into the underlying pathophysiological mechanisms, the development of CIPN is currently not preventable. Thus, the increasing incidence of CIPN is a highly relevant and growing clinical issue that leads to dose reduction, changes to less effective chemotherapeutic agents or even cessation of the therapy resulting in suboptimal cancer treatment (Areti et al., 2014). This review focusses on the commonly used antineoplastic substances oxaliplatin, cisplatin, vincristine, docetaxel, and paclitaxel which interfere with the cancer cell cycle, leading to cell death and tumor degradation, and cause severe acute and chronic peripheral neuropathies.

## Cancer chemotherapy: drug mechanism of action and metabolites

**Figure 1:**
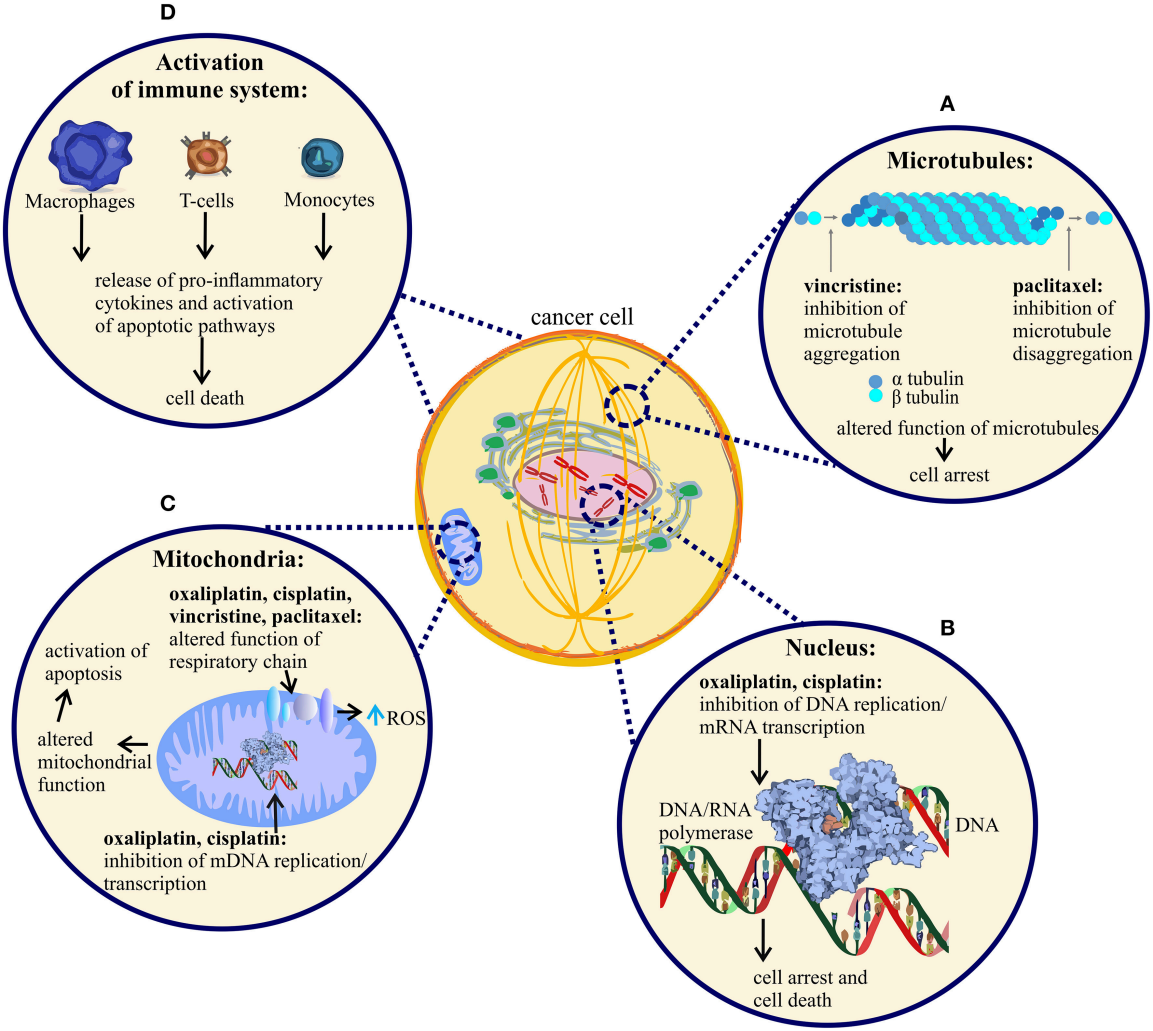
Mechanism of action of vincristine, paclitaxel, oxaliplatin and cisplatin. Anti-tumor mechanism of action of vincristine, paclitaxel, oxaliplatin and cisplatin leading to cell arrest and cell death. **(A)** Vincristine prevents microtubule aggregation, whereas paclitaxel prevents microtubule disaggregation, an effect leading to cancer cell division arrest and cell death. **(B)** Oxaliplatin and cisplatin bind to nuclear DNA (deoxyribonucleic acid) of cancer cells, causing disruption of DNA replication and RNA (ribonucleic acid) transcription and subsequent arrest of cancer cell division. The DNA adducts activate apoptotic pathways that induce cell death and tumor degradation. **(C)** All four anti-tumor agents alter the function of mitochondria, followed by disruption of respiratory chain function and increased production of reactive oxygen species (ROS). Additionally, oxaliplatin and cisplatin cause damage to cancer cell mitochondria by binding to mitochondrial DNA, altering mDNA replication and transcription. **(D)** All four agents cause activation of immune cells, an effect likely contributing to tumor cell degradation. Only a few representative immune cell-types are shown.

The mechanism of action of chemotherapeutic agents that lead to potent effects on tumor cell proliferation and cell death are well-studied and relatively well understood (Figure 1). However, it is not entirely clear whether these (mostly) desirable effects on rapidly proliferating cells are also responsible for causing undesirable effects on non-proliferating sensory neurons, or whether additional pharmacological effects contribute to the development of CIPN. While many chemotherapeutic agents can cause peripheral neuropathy, this is not a universal feature of all such compounds, suggesting that at least some additional mechanisms likely contribute. For example, carboplatin affects predominantly the hematopoeic system, while cisplatin and oxaliplatin both cause CIPN, albeit with different symptomatology. Similarly, while all vinca alkaloids can cause CIPN, this side effect is most common with vincristine, and less common with vinblastine, vinflunine, and vinorelbine (Grisold et al., 2012). These effects cannot be solely explained by different drug potencies, metabolites and pharmacokinetic properties. Nonetheless, the pathophysiology of CIPN is likely multi-factorial and contribution of at least some specific anti-cancer mechanisms, discussed below, is probable.

### Oxaliplatin and cisplatin

The platinum-based chemotherapeutic agents oxaliplatin and cisplatin, both of which are listed on the World Health Organization's List of Essential Medicines, are used for the treatment of various solid tumors. Oxaliplatin is used in combination with folinic acid and 5-fluorouracil as a part of the FOLFOX regimen for first-line and adjuvant colorectal cancer therapy, while cisplatin is one of the most effective treatments for solid tumors including small cell lung cancer, testicular, ovarian, brain, and bladder cancer. The intracellular concentration of platinum-based antineoplastics is maintained via several active transporters, including the copper transporter CTR1 which mediates drug uptake (Song et al., 2004) as well as copper-transporting ATPases that mediate efflux, such as ATP7A and ATP7B (Safaei and Howell, 2005).

Like other platinum-based compounds, oxaliplatin and cisplatin interfere with tumor cell proliferation via the formation of deoxyribonucleic acid (DNA)-platinum adducts. Cisplatin is converted to a strong electrophile after hydrolysis of its two chloride atoms, which are displaced by two molecules of water once the platinum complex enters the cell. Activated cisplatin then crosslinks two purine bases (adenine/guanine) of DNA by reacting with nitrogens at position seven of the purine rings, thus interfering with cell division and transcription of messenger ribonucleic acid (mRNA) (Dasari and Tchounwou, 2014). Similarly, the mono- and di-aquated oxaliplatin creates DNA intra-strand crosslinks by binding two guanine bases or a guanine-adenine pair of GC-rich regions of DNA (Faivre et al., 2003). Additionally, oxaliplatin causes inter-strand crosslinks and DNA-protein crosslinks which may contribute to the mode of action of oxaliplatin (Zwelling et al., 1979). These DNA mono- and bi-adducts in turn inhibit DNA replication and transcription, a fatal effect on cells in the S-phase of the cell cycle (when chromosomes are replicated; Alcindor and Beauger, 2011). Oxaliplatin also inhibits DNA transcription/mRNA production by interacting with transcription factors, inhibition of RNA polymerases and by creating nucleosomal DNA adducts leading to cell death (Todd and Lippard, 2009). In addition, activation of the immune system may be involved in the anticancer effects of oxaliplatin, with production of interferon γ by T-cells and resultant immunogenic cell death via TLR4 activation observed in both murine and human colon cancer cells (Tesniere et al., 2010). Likewise, cisplatin causes additional non-genomic effects including the production of reactive oxygen species resulting in altered mitochondrial function and activation of both intrinsic and extrinsic apoptosis pathways. Cisplatin also affects calcium signaling pathways and the function of several protein kinase families, including the MAPK (mitogen activated protein kinases), JNK (c-Jun N-terminal kinase o), PKC (protein kinase C) and AKT (serine/threonine kinases) leading to cell death (Dasari and Tchounwou, 2014).

A unique feature of oxaliplatin is its rapid non-enzymatic transformation to the reactive dichloro 1,2-diaminocyclohexyl-platinum complex and oxalate which occurs *via* replacement of the oxalate moiety with chloride ions in the blood plasma. Indeed, the generation of the oxalate metabolite is one of the few features distinguishing oxaliplatin from cisplatin, and has been proposed as one mechanism accounting for the differences in clinical presentation—most notably the presence of cold-induced neuropathy after treatment with oxaliplatin, but not cisplatin. However, a contribution of oxalate to oxaliplatin-induced cold pain has not been shown consistently (Deuis et al., 2013), and it is plausible that additional metabolites—including platinum complexes that bind to cellular proteins—may contribute to the development of CIPN.

### Vincristine

Vincristine is one of the most important antineoplastic substances used for chemotherapy of several childhood and adult tumors, and is—administered by intravenous infusion—a component of several different chemotherapy regimens (e.g., MOPP, COPP and BEACOPP regimen for therapy of Hodgkin's disease). While the mechanisms of vincristine transport into the cell are still unclear, a carrier-mediated transport mechanism characterized by Michaelis-Menten kinetics, temperature dependence and competitive inhibition was demonstrated in different murine leukemia cells (Bleyer et al., 1975). The adenosine triphosphate (ATP) binding cassette (ABC) transporter family, ABCB1, ABCC1, ABCC2, and ABCC3 contribute to vincristine efflux from the cell cytosol and to the development of cancer cell resistance to the therapy (Huang et al., 2006). Additionally, a relationship between cholesterol and phospholipid levels and vincristine uptake into murine leukemia cells has been demonstrated, showing that increased levels of cholesterol and phospholipids in the cell membrane accounts for lower vincristine accumulation (Pallares-Trujillo et al., 1993).

Vincristine binds to the β-subunit of tubulin and inhibits microtubule formation. The microtubules are cytoskeletal proteins that are involved in several important cell functions, for instance the regulation of cell shape, mitosis, chromosome segregation, cell division and retrograde as well as anterograde cellular transport. The proper function of microtubules depends on a balance between permanent aggregation and disaggregation of the α- and β-tubulin subunits. Therefore, the disruption of microtubule aggregation by bound vincristine can lead to mitotic arrest and cell death (Gan et al., 2010). The structure of the catharnine ring of the vinca alkaloids seems to be important for binding to axonal and cytoskeletal microtubules. Vinorelbine, composed of an eight-membered catharnine ring, shows a preference in binding to mitotic spindles over axonal microtubules, resulting in decreased neurotoxicity compared to vincristine, which is composed of a nine-membered catharnine ring (Gregory and Smith, 2000).

### Paclitaxel and docetaxel

Paclitaxel and docetaxel belong to the family of taxanes; chemotherapeutic agents used in the treatment of breast, prostate, lung, pancreatic, gynecological and other solid tumors that act by inhibiting disassembly of tubulin from the microtubule polymer. However, despite a similar mechanism of action, subtle differences in their molecular pharmacology, pharmacokinetics and pharmacodynamics result in distinct clinical effects. Docetaxel binds to tubulin with greater affinity than paclitaxel, and causes cell cycle arrest in the S rather than G2-M phase junction as is the case for paclitaxel (Dorr, 1997). Both compounds do not cross the blood-brain-barrier (BBB), although paclitaxel is accumulated in dorsal root ganglion neurons—which lie outside the BBB—via largely unknown mechanisms (Cavaletti et al., 1997, 2000; ten Tije et al., 2004; Wozniak et al., 2016). Both paclitaxel and docetaxel are extensively metabolized, via cytochrome P 2C8 and 3A4, respectively, albeit pharmacological activity of these metabolites is modest at best and their contribution to the development of CIPN is unknown.

### Cancer chemotherapy-induced peripheral neuropathy: epidemiology and clinical presentation

Chemotherapy-induced neuropathy is a common, dose-dependent side effect of many antineoplastics that not only leads to dose reduction or discontinuation of treatment, but also severely reduces the quality of life of patients (Bodurka-Bevers et al., 2000; Grisold et al., 2012). While the incidence of CIPN is compound-specific (Seretny et al., 2014), it also depends on patient co-morbidities and can, presumably, increase with concomitant treatment with other neurotoxic drugs (Johnson et al., 2015), although this effect has not been systematically investigated. Invariably, the percentage of patients suffering from CIPN as well as the severity of the condition increases with the (cumulative) dose (Dougherty et al., 2004; Seretny et al., 2014). Accordingly, the incidence of CIPN approaches nearly 100% for some agents at higher doses (Seretny et al., 2014), although differences in the definition, evaluation and reporting of peripheral neuronal deficits can lead to large variability in the reported occurrence. Notably, many studies rely on detailed clinician-administered grading scales or patient-reported outcomes using a range of questionnaires developed to evaluate the development of CIPN, while more objective assessments such as nerve conduction studies, nerve excitability studies, or quantitative sensory testing are used more rarely (Park S. B. et al., 2013). Importantly, CIPN is viewed by many clinicians as an unavoidable side effect of cancer chemotherapy, and one that is acceptable in light of the often greatly extended life-span offered by these chemotherapeutic agents (Cavaletti et al., 2011). In contrast, patients often view CIPN as a particularly troublesome side effect of cancer treatment that interferes significantly with their quality of life (Jones et al., 2015).

Clinically, CIPN presents as deficits in sensory, motor, and autonomic function which develop in a compound-specific manner (Park S. B. et al., 2013). Sensory symptoms usually develop first in the feet and hands—reflective of the axon length, with longer neurites affected first—and manifest as numbness, tingling, paresthesias and dysesthesias induced by touch, warm or cool temperatures, impaired vibration and altered touch sensations. In addition, painful sensations including non-evoked burning, shooting or electric shock-like pain as well as mechanical or thermal allodynia or hyperalgesia frequently occur (Postma et al., 1993; Sahenk et al., 1994; Becouarn et al., 1998; Bernhardson et al., 2007). In severe cases, these symptoms can progress to loss of sensory perception, which can be disabling (Strumberg et al., 2002). Motor symptoms include distal weakness, gait and balance disturbances and impaired fine movements. While rarer than sensory symptoms, motor impairment can progress to paralysis and cause significant functional disruption (Hile et al., 2010; Mols et al., 2016). In contrast, autonomic symptoms occur less frequently and may involve orthostatic hypotension, constipation and altered sexual or urinary function (Mols et al., 2016). With the exception of paclitaxel and oxaliplatin, which cause an acute neuropathy that emerges either during or shortly after infusion (Loprinzi et al., 2011; Argyriou et al., 2013b), the onset of CIPN is usually delayed and appears to depend on the total cumulative dose (Maestri et al., 2005). Some chemotherapeutics cause “coasting” of symptoms, the progressive worsening of neuropathy after cessation of treatment, and in severe cases CIPN can develop into an irreversible sensory neuron deficit.

### Cisplatin

Cisplatin causes a wide range of side effects including ototoxicity (hearing loss and tinnitus), nephrotoxicity, myelotoxicity and neuropathy. One of the most dose-limiting of these is peripheral neuropathy, which occurs in a dose- and time-dependent manner (Ozols and Young, 1985). The onset of cisplatin-induced neuropathy is variable, with some patients reporting the first appearance of symptoms after the first dose, and others after 12 cycles of therapy (Cersosimo, 1989; Vanderhoop et al., 1990). Generally, cisplatin-induced neuropathy develops after cumulative doses above 350 mg/m^2^, with approximately 92% of patients developing neurotoxic symptoms—characterized by tingling, numbness and mechanical and thermal hyperalgesia—at cumulative doses of 500–600 mg/m^2^ cisplatin (Roelofs et al., 1984; Thompson et al., 1984; Krarup-Hansen et al., 2007). Cisplatin-induced CIPN affects mostly the lower and upper limbs and includes mixed sensory and motor effects, including loss of vibration sense and taste, paresthesia, weakness, and tremor (Lomonaco et al., 1992; Amptoulach and Tsavaris, 2011). Motor nerve conduction velocity measurements in patients with decreased vibration sensitivity, loss of ankle jerks and paresthesias showed loss of sural nerve responses (Thompson et al., 1984), with emergence of reduced sensory nerve action potentials and loss of large myelinated fibers at higher cisplatin doses (Krarup-Hansen et al., 2007). The symptoms of cisplatin-induced neuropathy may persist for several months and can progressively worsen over time, a phenomenon called “coasting” (Siegal and Haim, 1990). With higher cumulative doses and longer times of exposure to cisplatin, the severity of CIPN increases, as does the likelihood of development of a chronic, irreversible neuropathy (Cersosimo, 1989; Gregg et al., 1992). Chronic cisplatin-induced neuropathy—the development of which appears to be independent of pre-treatment, vibration perception threshold, age, sex, tumor type or co-treatment with other chemotherapeutics—has been reported in approximately 5–20% of patients at 12 months (Hilkens et al., 1994; Hoskins et al., 1994; Bogliun et al., 1997; Schmoll et al., 2003; Park S. B. et al., 2013).

**Figure 2:**
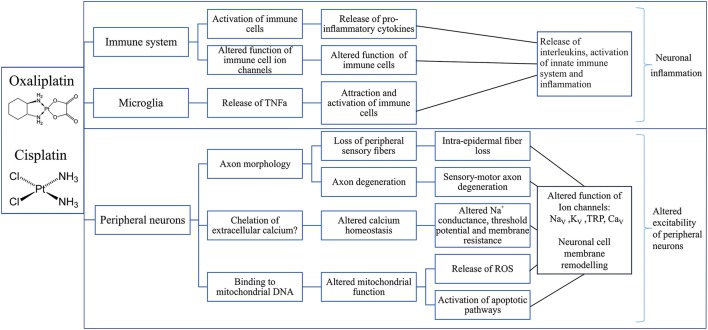
Putative mechanisms involved in the development of cisplatin- and oxaliplatin-induced peripheral neuropathy. Overview of possible effects of oxaliplatin and cisplatin on the immune system (Raghavendra et al., 2003; Callizot et al., 2008; Boyette-Davis and Dougherty, 2011; Wang et al., 2012; Di Cesare Mannelli et al., 2014; Janes et al., 2015), microglia (Di Cesare Mannelli et al., 2014) and peripheral neurons (Zwelling et al., 1979; Thompson et al., 1984; Faivre et al., 2003; Tomaszewski and Busselberg, 2007; Todd and Lippard, 2009; Tesniere et al., 2010; Alcindor and Beauger, 2011; Boyette-Davis and Dougherty, 2011; Deuis et al., 2013; Areti et al., 2014; Boehmerle et al., 2014; Dasari and Tchounwou, 2014; Canta et al., 2015; Leo et al., 2017) leading to neuronal inflammation (Janes et al., 2015) and altered neuronal excitability (Adelsberger et al., 2000; Krishnan et al., 2005; Kagiava et al., 2008; Gauchan et al., 2009; Ta et al., 2010; Descoeur et al., 2011; Nassini et al., 2011; Schulze et al., 2011; Sittl et al., 2012; Deuis et al., 2013, 2014; Yamamoto et al., 2015; Mizoguchi et al., 2016). TNFα, Tumor necrosis factor alpha; DNA, deoxyribonucleic acid; ROS, Reactive oxygen species; Na_V_, Voltage-gated sodium channel; K_V_, Voltage-gated potassium channel; TRP, Transient receptor potential channel; Ca_V_, Voltage-gated calcium channel.

Several mechanisms contributing to cisplatin-induced neurotoxicity have been suggested (Figure 2), including the loss of peripheral sensory neurons, changes in cell signaling cascades, changes to calcium homeostasis and signaling, oxidative stress, mitochondrial dysfunction and induction of apoptosis as a result of DNA platination (Meijer et al., 1999).

### Oxaliplatin

Treatment with oxaliplatin induces various side effects including myelotoxicity and peripheral neuropathy, albeit it lacks the ototoxic and nephrotoxic effects commonly observed after treatment with cisplatin. The neurotoxic effects of oxaliplatin include the development of an acute, transient neuropathy that occurs in almost 90% of patients within hours of infusion and is characterized by dysesthesias and paresthesias of the hands, feet and the perioral region. These symptoms are often induced by exposure to cool temperatures and are a key feature of oxaliplatin-induced cold allodynia (Argyriou et al., 2013a). In addition, motor symptoms including tetanic spasms, fasciculations, and prolonged muscular contractions commonly occur (Saif and Reardon, 2005). Acute oxaliplatin-induced neuropathy generally subsides between treatment cycles (Extra et al., 1998), although continued exposure to oxaliplatin can lead to the development of a severe chronic neuropathy. The incidence of chronic peripheral neuropathy following oxaliplatin treatment has been estimated as approximately 70%, with development of the condition usually occurring at cumulative doses exceeding 540 mg/m^2^ (Cersosimo, 2005; Argyriou et al., 2013a). Clinically, the symptoms of chronic oxaliplatin-induced neuropathy closely resemble those of the acute condition and include temperature-insensitive paresthesias, hypoesthesias and dysesthesias of the hands and feet. Additionally, changes in proprioception, which may affect normal daily activities requiring fine motor coordination, reportedly occur at cumulative doses exceeding 780 mg/m^2^ (Cersosimo, 2005; Saif and Reardon, 2005).

Oxaliplatin-induced central neuropathy is rare and is characterized by Lhermitte's sign (an electric sensation experienced with flexing of the neck), proprioception deficiencies and urinary retention (Cersosimo, 2005). Risk factors for the development of both acute and chronic neuropathy include the cumulative dose, low body weight, a body-surface area >2.0, young age, persistent neuropathy in a past cycle, and variations in genes such as glutathione-S-transferase genes P1 [GSTP1] and glutathione-S-transferase genes M1 [GSTM1], as discussed below (Saif and Reardon, 2005; Alejandro et al., 2013). Furthermore, oxaliplatin induced neuropathy may be exacerbated by surgery (Gornet et al., 2002).

The mechanisms contributing to development of oxaliplatin-induced CIPN (Figure 2) include alteration in axonal excitability due to ion channel dysfunction, dysregulation of calcium homeostasis and altered function of transient receptor potential channels. Additionally, oxidative stress leading to neuronal and glial cell dysfunction and cell death induced by caspases, mitogen-activated protein kinases and protein kinase C may contribute to development of oxaliplatin-induced CIPN (Carozzi et al., 2015).

### Vincristine

Despite widespread use of vincristine for the treatment of pediatric cancers including acute lymphoblastic leukemia, sarcoma, medulloblastoma and neuroblastoma as well as a range of tumors in adults, the epidemiology of vincristine-induced peripheral neuropathy (VIPN) is relatively poorly defined. While the reported incidence rates vary considerably—depending on the severity of symptoms, diagnostic criteria and concomitant use of other neurotoxic agents—most patients receiving vincristine develop some degree of sensory peripheral neuropathy at a cumulative dose of >4 mg/m^2^ (Ramchandren et al., 2009; Toopchizadeh et al., 2009; Seretny et al., 2014; Lavoie Smith et al., 2015; Tay et al., 2017).

Symptoms of neuropathy can be present soon after initiation of vincristine therapy but usually develop within several weeks, and generally worsen with increasing cumulative doses of the drug. In addition, genetic factors can enhance the susceptibility to develop vincristine-induced neuropathy. An increased severity of VIPN was also observed in older compared with younger children, suggesting that age can be a risk factor for development of VIPN (Lavoie Smith et al., 2015).

VIPN can be divided into sensory, motoric and autonomic components, with tendon reflexes, vibration sensitivity and strength most affected in the first year of treatment (Lavoie Smith et al., 2015). Vincristine-induced sensory neuropathy is characterized by numbness, tingling and neuropathic pain in the upper and lower extremities. In addition, patients receiving vincristine experience loss of sensory discrimination, specifically an inability to detect light touch, pinprick sensations or vibration, and an inability to differentiate between hot and cold temperatures (Barton et al., 2011). Vincristine-induced motor neuropathy is characterized by weakness in the upper and lower extremities and the development of wrist- or foot-drop due to impaired dorsiflexion that arises from damage to peripheral nerves. This is accompanied by gait abnormalities, cramps, and loss of or reduction in deep tendon reflexes which can be severe (Mora et al., 2016). Typical symptoms of autonomic neuropathy are constipation, urinary retention, and orthostatic hypotension. As would be expected, these symptoms can significantly reduce the quality of life of these patients (Lavoie Smith et al., 2013).

**Figure 3:**
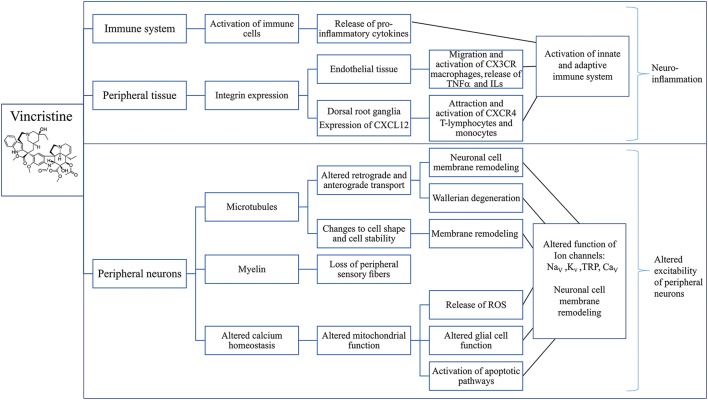
Putative mechanisms involved in the development of vincristine-induced peripheral neuropathy. Overview of possible effects of vincristine on the immune system (Callizot et al., 2008; Kiguchi et al., 2008; Wang et al., 2012; Chatterjee et al., 2014; Old et al., 2014; Makker et al., 2017), peripheral tissues (Old et al., 2014) and sensory neurons (Kaba et al., 1985; Topp et al., 2000; Gan et al., 2010; Areti et al., 2014; Canta et al., 2015; Carozzi et al., 2015; Xu et al., 2017) leading to neuronal inflammation (Chatterjee et al., 2014; Xu et al., 2017) and altered excitability of peripheral neurons (Alessandri-Haber et al., 2008; Old et al., 2014) which may be considered as the main mechanisms contributing to the development of vincristine-induced CIPN. CXCL12, C-X-C Motif Chemokine Ligand 12; CX3CR, C-X-3-C motif chemokine receptor; TNF α, Tumor necrosis factor alpha; ILs, Interleukins; CXCR4, C-X-C motif chemokine receptor 4; ROS, Reactive oxygen species; Na_V_, Voltage-gated sodium channel; K_V_, Voltage-gated potassium channel; TRP, Transient receptor potential channel; Ca_V_, Voltage-gated calcium channel.

The mechanisms contributing to development of VIPN include disruption of calcium homeostasis, activation of the immune system and subsequent neuroinflammation, membrane remodeling of peripheral neurons and loss of large myelinated fibers (Figure 3) (Devor, 2006; Boehmerle et al., 2014; Carozzi et al., 2015).

### Taxanes (paclitaxel, docetaxel)

CIPN is also a dose-limiting adverse effect of treatment with taxanes, particularly paclitaxel, and occurs in a dose- and treatment duration-dependent manner. The threshold dose for development of taxane-induced peripheral neuropathy lies close to standard doses used in a range of chemotherapy regimens at approximately 300 mg/m^2^ for paclitaxel, and 100 mg/m^2^ for docetaxel (Forsyth et al., 1997; Winer et al., 2004; Park S. B. et al., 2013). Accordingly, this symptom is very common, occurring in as many as 90% of patients, although it usually remains relatively mild until cumulative paclitaxel doses exceed 1,400 mg/m^2^ (Lipton et al., 1989; van Gerven et al., 1994; Pace et al., 2007). Overall, while paclitaxel is slightly less potent than docetaxel, it is more commonly associated with the development of CIPN (Hilkens et al., 1997; Chon et al., 2009). Consistent with CIPN being a direct consequence of the pharmacological effects of the taxanes on sensory neurons, higher single as well as higher cumulative doses are associated with both a greater risk for development of this side effects, as well as increased severity of symptoms (Pace et al., 2007; Baldwin et al., 2012; Ghoreishi et al., 2012). In addition, concomitant treatment with other neurotoxic chemotherapeutic agents, or pre-existing neuropathies, may additionally increase the risk of developing CIPN (Chaudhry et al., 2003). While symptoms usually develop within several weeks of treatment initiation, paclitaxel and docetaxel can also induce an acute painful neuropathy—peaking approximately 3 days after infusion—that is characterized by pain, numbness and tingling, and which can be a predictor for the development of chronic neuropathy (Loprinzi et al., 2007, 2011; Reeves et al., 2012; Tanabe et al., 2013; Fernandes et al., 2016).

Symptoms of taxane-induced neuropathy are consistent with a predominantly sensory neuropathy, although motor effects (particularly distal weakness, muscle cramps, and muscle aches) and autonomic dysfunction (including arrhythmias and orthostatic hypotension) can occur with higher doses. Specifically, numbness, tingling, mechanical allodynia, and neuropathic pain developing symmetrically in the digits and extending to the extremities usually predominate; cold allodynia, loss of pinprick sensation as well as altered reflexes can also occur (Dougherty et al., 2004; Park et al., 2011; Tofthagen et al., 2013).

**Figure 4:**
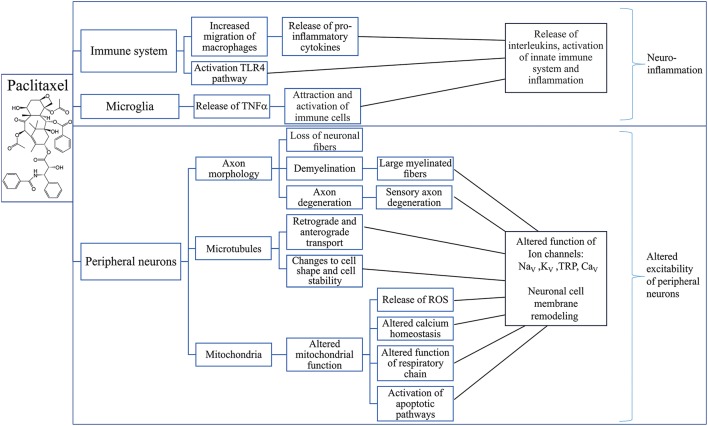
Putative mechanisms involved in the development of paclitaxel-induced peripheral neuropathy. Overview of possible effects of paclitaxel on the immune system (Ledeboer et al., 2007; Loprinzi et al., 2007, 2011; Callizot et al., 2008; Doyle et al., 2012; Wang et al., 2012; Zhang et al., 2012, 2016; Pevida et al., 2013; Janes et al., 2014a,b; Liu et al., 2014; Li et al., 2015; Krukowski et al., 2016; Zhang et al., 2016; Makker et al., 2017), microglia (Burgos et al., 2012; Ruiz-Medina et al., 2013; Makker et al., 2017) and peripheral neurons (Sahenk et al., 1994; Cavaletti et al., 1997, 2000; Dorr, 1997; Kidd et al., 2002; ten Tije et al., 2004; Mironov et al., 2005; Flatters and Bennett, 2006; Argyriou et al., 2008; Doyle et al., 2012; Areti et al., 2014; Boehmerle et al., 2014; Griffiths and Flatters, 2015; Duggett et al., 2016; Wozniak et al., 2016) leading to neuronal inflammation (Ledeboer et al., 2007; Loprinzi et al., 2007, 2011; Callizot et al., 2008; Doyle et al., 2012; Wang et al., 2012; Zhang et al., 2012, 2016; Pevida et al., 2013; Janes et al., 2014a,b; Liu et al., 2014; Li et al., 2015; Krukowski et al., 2016; Zhang et al., 2016; Makker et al., 2017) and altered excitability of peripheral neurons (Materazzi et al., 2012; Zhang and Dougherty, 2014). TLR4, Toll-Like Receptor 4; TNFα, Tumor necrosis factor alpha; ROS, Reactive oxygen species; Na_V_, Voltage-gated sodium channel; K_V_, Voltage-gated potassium channel; TRP, Transient receptor potential channel; Ca_V_, Voltage-gated calcium channel.

Mechanisms that contribute to paclitaxel-induced neuropathy include immune-mediated processes, loss of peripheral fibers, demyelination and axon degeneration, altered retrograde and anterograde transport as well as mitochondrial dysfunction (Figure 4).

## The genetics of chemotherapy-induced peripheral neuropathy

In recent years, several studies have identified genetic risk factors associated with the development of CIPN in cancer patients. Many of these are pharmacogenomic in nature, affecting either the absorption, distribution, metabolism or excretion of these chemotherapeutic agents. For instance, polymorphisms in glutathione transferases, cytochrome P450 enzymes and ATP binding cassette transporters may be involved in the development of varying types of CIPN as they affect the uptake and disposition of various cytotoxic drugs (Broyl et al., 2010; Johnson et al., 2011).

Specifically, the polymorphism Ile105Val of the GSTP1 gene, encoding glutathione transferase P1, has been associated with a decreased risk of developing severe oxaliplatin-related cumulative neuropathy. This mutation is thought to increase the activity of glutathione transferase P1, an enzyme that catalyzes the conjugation of hydrophobic and electrophilic compounds with glutathione, thus reducing the toxicity level of the bound substances. Indeed, *in vitro* experiments in *Escherichia coli* carrying different GSTP1 mutations demonstrated altered cytotoxicity, suggesting that GSTP1 polymorphisms could be predictors for the development of cumulative neuropathy (Ishimoto and Ali-Osman, 2002; Lecomte et al., 2006). Similarly, polymorphisms in GSTM1, the gene encoding the enzyme Glutathione S-Transferase Mu 1, have been associated with a lower incidence of cisplatin-induced neuropathy (Khrunin et al., 2010), while polymorphism of the CYP450 3A enzyme system can necessitate vincristine dose adjustments to prevent neurotoxicity (Mora et al., 2016).

An important contributing factor to the development of CIPN is thought to be the cellular uptake and accumulation of platinum-derivatives in sensory neurons (Liu et al., 2009, 2012, 2013). Specifically, the copper transporter 1 (CTR1), members of the organic cationic transporter family (OCT), and copper-transporting ATPases have been proposed as key transporters maintaining the intracellular concentration of platinum derivatives *via* active uptake and efflux processes (Holzer et al., 2006; Liu et al., 2012; Sprowl et al., 2013; Cavaletti et al., 2014). Several of these transporters are also expressed on the plasma membrane of dorsal root ganglion cells, where they presumably contribute to the development of CIPN. While the human CTR1 plays a particularly important role in both resistance to platinum drugs and uptake in sensory neurons *in vivo* and *in vitro* (Song et al., 2004; Liu et al., 2009, 2013), residual oxaliplatin accumulation in murine embryonic fibroblast from CTR1^−/−^ animals suggests the existence of additional transport mechanisms leading to accumulation of platinum-based compounds (Holzer et al., 2006). These likely include the organic cationic transporters belonging to the solute carrier family. In particular, OCT1 (SLC22A1) and OCT2 (SLC22A2) and the cation and carnitine transporters OCTN1 (SLC22A4) and OCTN2 (SLC22A5) have been suggested to be involved in the cellular uptake of platinum drugs (Ceresa and Cavaletti, 2011). OCT2 in particular contributes significantly to accumulation of oxaliplatin, and genetic or pharmacological knockout of OCT2 prevented the development of cold and mechanical hypersensitivity following treatment with oxaliplatin, suggesting that the OCT2 transporter plays a crucial role in oxaliplatin-induced cytotoxicity (Sprowl et al., 2013).

Notably, a polymorphism in the ABCC2 gene has also been suggested to lead to higher oxaliplatin concentration in neurons, and was associated with oxaliplatin induced neuropathy (Mori et al., 2008). Two additional SNPs in the same gene, rs3740066 GG and rs12826 GG, have been associated with an increased risk to develop neuropathy following vincristine treatment, presumably due to increased accumulation of vincristine in neuronal cell bodies leading to increased neurotoxicity (Lopez-Lopez et al., 2016). In contrast, ABCB1, CYP1B1, and CYP2C8 gene mutations were associated with paclitaxel-induced neuropathy (Boora et al., 2016).

In addition to genetic factors affecting the pharmacokinetics of chemotherapeutic agents, several polymorphisms relating to pharmacodynamic effects have been causally implicated in the development of CIPN. These include genetic risk factors that alter sensitivity to cytotoxic effects, cellular repair mechanisms, and excitability of sensory neurons. For example, polymorphisms that alter the expression of the DNA excision repair protein ERCC-1 (excision repair cross-complementing group 1) have been suggested to be involved in cisplatin- and oxaliplatin-induced cytotoxicity, though no clinical study to date has shown an association with CIPN (Inada et al., 2010). Genetic variants of alanine glyoxylate aminotransferase (AGXT), an enzyme involved in oxalate metabolism, have been correlated with the severity of oxaliplatin-induced CIPN (Gamelin et al., 2007). Similarly, a polymorphism in the ITGB3 gene—encoding for Integrin B3—was not correlated with the development of oxaliplatin-induced CIPN, although it appeared to be related to the severity of this symptom (Antonacopoulou et al., 2010). This effect may be mediated *via* the differential activation of the mitogen activated protein kinases MAPK3 and MAPK1 that are induced by these ITGB3 variants, and which may contribute to development of CIPN (Scuteri et al., 2009). Altered development of acute oxaliplatin-induced peripheral neuropathy was also reported in patients with single nucleotide polymorphisms (SNPs) in SCNA genes encoding the voltage-gated sodium channels, which are essential for the initiation and propagation of action potential in neurons. Specifically, polymorphisms in the Na_V_1.4 (SCN4A-rs2302237) and Na_V_1.8 genes (SCN10A-rs1263292) have been associated with increased incidence of acute oxaliplatin-induced neuropathy (Argyriou et al., 2013a), while patients carrying a polymorphism (rs6746030) in the SCN9A gene encoding for Na_V_1.7 seem to develop less severe neuropathy than patients carrying other SCN9A gene variants (Sereno et al., 2017).

Genetic variants associated with the development and severity of taxane-induced neuropathy include low-frequency variants in the ephrin receptor genes EPHA6, EPHA5, and EPHA8 (Leandro-Garcia et al., 2013; Apellaniz-Ruiz et al., 2017), the Charcot-Marie-Tooth disease gene ARHGEF10 (Boora et al., 2015), the glycogen synthase kinase-3β (GSK3β) gene (Park et al., 2014), the DNA repair pathway genes XPC (Lamba et al., 2014), the congenital peripheral neuropathy gene FGD4 (Baldwin et al., 2012), the β-tubulin IIa gene (TUBB2A) (Leandro-Garcia et al., 2012) and VAC14, a gene coding for a component of a trimolecular complex that tightly regulates the level of phosphatidylinositol 3,5-bisphosphate (Hertz et al., 2016).

A SNP in the centrosome protein encoded by CEP72 gene has been associated with development of vincristine-induced neuropathy in the later phases of therapy. This protein is important for microtubule formation and appears to increase the sensitivity of neuronal cells to vincristine damage (Diouf et al., 2015). Additionally, several other SNPs, including in CAMKK1 (Calcium/Calmodulin Dependent Protein Kinase 1, involved in regulation of apoptosis), CYP2C8 (Cytochrome P450 Form 1) and CYP2C9 (Cytochrome P450 PB-1, both involved in hepatic drug clearance), NFATC2 (Nuclear Factor of Activated T-Cells 2), ID3 (Inhibitor Of Differentiation 3) and SLC10A2 (apical sodium-dependent bile acid transporter) have been suggested to be involved in vincristine-induced neuropathy (Johnson et al., 2011).

## Pathological mechanisms contributing to CIPN

The pathological mechanisms underlying the development of CIPN have been studied extensively, and likely involve direct effects on the viability of sensory neurons, as well as cell-type specific consequences that occur downstream of on-target pharmacological activity of these cytotoxic drugs. In addition, off-target effects and additional mechanisms may also be involved, although the relative causative contribution remains unclear. Overall, despite diverse pharmacological mechanisms, a number of common pathologies have been proposed, including oxidative stress, altered calcium homeostasis, axon degeneration, membrane remodeling, and inflammatory processes (Figures 2–4).

### Oxidative stress and apoptotic pathways

Mitochondria are small organelles involved in many important cellular processes including energy production, storage of intracellular calcium and calcium signaling, apoptosis, regulation of membrane potential and cell metabolism. The main function of mitochondria is to produce adenosine triphosphate (ATP) *via* aerobic respiration. In healthy tissues, mitochondria produce small amounts of reactive oxygen species (ROS) such as peroxide, superoxide, hydroxyl radicals and singlet oxygen as a by-product of oxygen metabolism. These radicals carry out important functions in cell signaling.

Most chemotherapeutic agents cause damage to neuronal and non-neuronal mitochondria, leading to increased production of ROS and thus to increased oxidative stress (Sangeetha et al., 1990; Look and Musch, 1994; Weijl et al., 1998; McDonald and Windebank, 2002). The pathological increase in ROS production in turn can cause damage to intracellular biomolecules such as enzymes, proteins and lipid molecules (Slater, 1984; Stadtman et al., 2003; Valko et al., 2005), which in turn leads to demyelination and disruption of the cytoskeleton of peripheral nerves as well as sensitization of signal transduction processes (Anderson et al., 1992; Zheng et al., 2011). Furthermore, ROS can cause the activation of apoptotic pathways (Cashman and Hoke, 2015) and increase production of pro-inflammatory mediators (Bulua et al., 2011). These processes can cause further damage to mitochondria, amplifying the production of ROS and pathological processes of oxidative stress (Areti et al., 2014).

Oxaliplatin and cisplatin bind to mitochondrial DNA (mDNA) and form mDNA adducts which cannot be repaired since mitochondria do not express DNA repair systems. These Pt-mDNA adducts impair mitochondrial DNA replication and transcription, leading to altered protein synthesis and functional errors of the respiratory chain (Canta et al., 2015). Similarly, vincristine causes dysregulation and structural modification of neuronal mitochondria, leading to activation of apoptotic pathways, alteration in neuronal excitability and dysfunction of glial cells. In contrast to platinum-induced mitochondrial dysfunction, the effects of vincristine on mitochondria likely involves altered mitochondrial Ca^2+^ signaling (Canta et al., 2015; Carozzi et al., 2015). This hypothesis is supported by the observation that potentiation of vincristine-induced cytotoxicity occurs in the presence of verapamil, a calcium channel blocker (Kaba et al., 1985).

Like vincristine, paclitaxel does not directly affect mitochondrial DNA, but nonetheless induces swollen and vacuolated mitochondria in both myelinated and unmyelinated sensory axons in the saphenous nerve (Flatters and Bennett, 2006). These changes in turn are accompanied by increased ROS production in sensory neurons and spinal cord, although the mechanisms leading to altered mitochondrial function are relatively poorly understood (Doyle et al., 2012; Areti et al., 2014; Griffiths and Flatters, 2015; Duggett et al., 2016).

### Calcium homeostasis

Calcium (Ca^2+^) is a key regulatory ion in many cellular and physiological processes. Its free intracellular concentration, which is usually maintained at nanomolar levels, is tightly regulated by various transport and sequestration mechanisms, including extracellular influx and release from internal stores, as well as efflux via plasma membrane pumps and uptake into the endoplasmic reticulum and mitochondria (Siau and Bennett, 2006). Changes in the intracellular Ca^2+^ concentration influence membrane excitability, neurotransmitter release and gene expression of neuronal and glial cells (Carozzi et al., 2015). Accordingly, dysregulation of Ca^2+^ homeostasis and Ca^2+^ signaling has been suggested to contribute to the development of oxaliplatin-, cisplatin-, vincristine-, and paclitaxel-induced CIPN.

The oxaliplatin metabolite oxalate is a well-known Ca^2+^ chelator that has been proposed to contribute to the development of oxaliplatin-induced CIPN. Indeed, local injection of oxalate into the footpad of mice induces spontaneous nocifensive behavior as well as mechanical allodynia, albeit the dose required to observe this effect is considerably higher than the dose of oxaliplatin required to induce neuropathic pain (Deuis et al., 2013). In addition, the phenotype of pain behaviors induced by oxalate differs significantly from oxaliplatin-induced neuropathy, which is characterized by cold allodynia and a lack of spontaneous nocifensive behavior (Deuis et al., 2013). Oxalate-induced pain likely arises as a consequence of chelation of extracellular Ca^2+^ ions, which in turn leads to an increase in Na^+^ conductance and a decrease of threshold potential and membrane resistance (Deuis et al., 2013). In contrast, an increase in extracellular Ca^2+^ concentration increases the probability of Na^+^ channel closure and results in decreased excitability of peripheral neurons (Armstrong and Cota, 1999). Accordingly, the administration of Ca^2+^/Mg^2+^ prior to oxaliplatin infusion has been evaluated in several clinical trials as a strategy to prevent development of CIPN, although a consistent clinical benefit is unfortunately not apparent (Jordan et al., 2016).

In contrast, although effects on intracellular Ca^2+^ levels have been reported both in sensory neurons, renal tubular cells and cancer cells, the contribution of Ca^2+^ to cisplatin-induced neuropathy is relatively poorly understood. In sensory neurons, cisplatin increased the expression of the N-type voltage-gated Ca^2+^ channels (Ca_V_2.2), although it differentially affects the function of specific Ca_V_ channel subtypes (Tomaszewski and Busselberg, 2007; Leo et al., 2017). However, the contribution of these effects to the clinical presentation of cisplatin-induced neuropathy remains unclear.

Contributions of Ca^2+^ signaling to the pathology of CIPN have also been reported for paclitaxel, which causes rapid mitochondrial depolarization and Ca^2+^ release in both neuronal and non-neuronal cells, possibly via activation of the mitochondrial permeability transition pore (mPTP) (Kidd et al., 2002; Mironov et al., 2005). In addition, lower concentrations of paclitaxel induce Ca^2+^ oscillations downstream of neuronal calcium sensor 1 (NCS-1), a protein that regulates G protein-coupled receptor phosphorylation in a calcium-dependent manner (Boehmerle et al., 2006).

### Axon degeneration

Several studies in humans and animals have demonstrated axon degeneration after long-term administration of chemotherapeutic agents, including the loss of large myelinated, small unmyelinated (more rarely), and intra-epidermal nerve fibers (IENF) which may be connected to the development of sensory-motoric peripheral neuropathy (Bradley et al., 1970; Cavaletti et al., 1992; Sahenk et al., 1994; Bennett et al., 2011; Boyette-Davis et al., 2011, 2013; Boehmerle et al., 2014). Intra-epidermal nerve fibers are unmyelinated or thinly myelinated nociceptors located in the dermis and are necessary for the sensation of pain arising from the periphery. Additionally, loss of myelin and changes to the axonal cytoskeleton likely alters the structure and function of peripheral nerves, which in turn may contribute to development of altered perception. However, while correlation of nerve fiber loss with the degree of neuropathy has been attempted in several conditions, and functional deficits arising from axon degeneration appear intuitive, the contribution of demyelination and peripheral nerve degeneration to the pathobiology of CIPN is not entirely clear.

Clinical and electrophysiological studies have shown that oxaliplatin causes moderate sensory-motor axon degeneration and loss of intra-epidermal nerve fibers (Boyette-Davis and Dougherty, 2011). Similarly, electron microscopy of peripheral nerves from cisplatin-treated patients revealed axonal degeneration of large myelinated fibers as well as secondary myelin breakdown associated with loss of ankle jerks and decreased vibration sensitivity (Thompson et al., 1984). In mice, cisplatin damaged myelinated fibers of the sciatic nerve, diminished the action potential amplitude and reduced the nerve conduction velocity of the caudal sensory nerve (Boehmerle et al., 2014). Degeneration of distal sensory axons, secondary demyelination and nerve fiber loss have also been reported in vincristine- and paclitaxel-induced neuropathy (Sahenk et al., 1994; Topp et al., 2000; Argyriou et al., 2008; Boehmerle et al., 2014).

The molecular mechanisms leading to these observed changes in axonal function and structure remain unclear. Chemotherapeutics causing CIPN likely have direct toxic effects on axons—evidenced by a lack of effect on axon integrity when applied to neuronal cell bodies under compartmentalized culturing conditions (Yang et al., 2009)—although indirect effects due to altered gene expression cannot be ruled out. Altered microtubule function can also impair the anterograde and the retrograde axonal transport of synaptic vesicles loaded with lipids, proteins and ion channels. This in turn causes a length-dependent degeneration of axonal distal segments (Wallerian degeneration) and axonal membrane remodeling of peripheral nerves. However, these effects cannot adequately explain axon degeneration induced by cisplatin and oxaliplatin, which target cellular DNA. In addition, the different vinca alkaloids used in chemotherapy display variable efficacies and side effects but equally block tubulin polymerization, suggesting that additional mechanisms contribute to the direct damage of peripheral nerves (Himes et al., 1976).

### Changes in neuronal excitability

In addition to axonal degeneration, chemotherapeutic agents also cause changes to peripheral nerve excitability that contribute to the development of sensory peripheral neuropathy. These are likely caused by altered expression and function of a range of ion channels—including voltage-gated sodium (Na_V_), voltage-gated potassium (K_V_) and transient receptor potential (TRP) channels.

Changes in sensory nerve excitability in patients treated with oxaliplatin, including a significant increase in the duration of the relative refractory period, have been attributed to effects on Na_V_ channels expressed at the nodes of Ranvier (Krishnan et al., 2005). Altered Na_V_ channel function was also observed in rodent peripheral axons as well as dorsal root ganglion neurons, where oxaliplatin causes an increase in Na^+^ current, inhibition of maximal amplitude, and the emergence of enhanced resurgent and persistent current amplitudes (Adelsberger et al., 2000; Sittl et al., 2012). Specifically, the Na_V_ channel isoform Na_V_1.6 appears to be involved in the development of oxaliplatin-induced cold allodynia, with cooling-induced bursts of action potential firing abolished in neurons from Scn8a(med/med) mice lacking functional Na_V_1.6 (Sittl et al., 2012). Furthermore, acute oxaliplatin-induced cold pain behaviors were abolished by treatment with a selective Na_V_1.6 inhibitor, as was cisplatin-induced mechanical allodynia, albeit cisplatin does not directly affect the gating properties of Na_V_1.6 (Deuis et al., 2013, 2014).

Effects on neuronal potassium (K^+^) channels—of which four major groups [voltage-gated K^+^ channels (K_V_), Ca^2+^-activated K^+^ channels (K_*Ca*_), two-pore K^+^ channels (K_2P_) and inwardly-rectifying K^+^ channels (K_ir_)] are expressed in peripheral nerves—further exacerbate altered neuronal excitability in CIPN. Specifically, decreased expression of several K^+^ channels, including the K_2P_ channels TREK1 and TRAAK, were found after oxaliplatin and paclitaxel treatment in rodent dorsal root ganglion neurons (Descoeur et al., 2011; Zhang and Dougherty, 2014). In addition, broadening of the repolarization phase, repetitive firing and after-hyperpolarization, consistent with effects on K_V_ channels, were induced by treatment with oxaliplatin in the isolated sciatic nerve of the adult rat (Kagiava et al., 2008).

In addition to acute effects on sensory neuron excitability, exposure to oxaliplatin and cisplatin has also been reported to alter expression of several thermo- and mechano-sensitive TRP channels, including TRPV1, TRPA1, and TRPM8 (Gauchan et al., 2009; Ta et al., 2010; Descoeur et al., 2011; Nassini et al., 2011; Schulze et al., 2011; Yamamoto et al., 2015; Mizoguchi et al., 2016). The reported behavioral contributions of these channels to CIPN, however, remain seemingly at odds, with studies attributing functional effects to all, some, or none of these transducer ion channels. Whether these discrepancies arise from insufficient subtype selectivity of compounds with activity at TRP channels, compensatory expression changes in knockout animals, or difference between animal models, remains to be determined. Paclitaxel- and vincristine-induced neuropathy has also been attributed to activation of TRPA1 and TRPV4 via the generation of reactive oxygen species (Alessandri-Haber et al., 2008; Materazzi et al., 2012; Old et al., 2014), albeit disruption of TRP channel function due to altered association with microtubules may be an additional contributing mechanism (Goswami, 2012).

### Activation of the immune system and inflammation

Chemotherapy agents are well known to cause profound effects on the immune system, most notably a transient immunosuppression due to inhibition of myeloproliferation. However, activation of the immune system by chemotherapeutics has increasingly received attention as an effect that is thought to support the destruction of tumor cells (Zitvogel et al., 2008; Westbom et al., 2015), but which may also lead to neuroinflammation and thus contribute to the development of CIPN. Specifically, effects of chemotherapeutics on the innate (Kiguchi et al., 2008; Liu et al., 2014; Li et al., 2016) and adaptive immune system (Zhang et al., 2008; Zhu et al., 2011; Krukowski et al., 2016), as well as effects on peripheral and central neuronal accessory cells—including satellite glial cells (Peters et al., 2007; Kiya et al., 2011; Warwick and Hanani, 2013), Schwann cells (Cavaletti et al., 1995), astrocytes (Zhang et al., 2012; Robinson et al., 2014) and microglia (Burgos et al., 2012; Ruiz-Medina et al., 2013)—have been observed, although most of these studies were carried out using rodent models (Lees et al., 2017).

Consistent with a contribution of the immune system to CIPN, oxaliplatin- and paclitaxel-induced mechanical hyperalgesia and epidermal nerve fiber loss was prevented by the tetracycline minocycline, an antibiotic known to inhibit macrophages/monocytes and microglia (Raghavendra et al., 2003; Liu et al., 2010; Boyette-Davis and Dougherty, 2011; Di Cesare Mannelli et al., 2014). Both chemotherapeutic agents also lead to increased levels of proinflammatory cytokines (IL-6, IL-8, IL1β, TNF-α), which can lead to sensitization of nociceptors (Ledeboer et al., 2007; Loprinzi et al., 2007, 2011; Callizot et al., 2008; Doyle et al., 2012; Wang et al., 2012; Zhang et al., 2012, 2016; Pevida et al., 2013; Janes et al., 2014a,b, 2015; Li et al., 2015; Makker et al., 2017). In addition, oxaliplatin increases the levels of circulating CD4+ and CD8+ lymphocytes in mice and down-regulates regulatory T (T-reg) cells (Makker et al., 2017).

Vincristine induces the expression of integrins (immune markers) on the surface of endothelial cells which allows macrophages expressing the CX3CR receptor to adhere to the endothelium and to migrate into nervous tissue. Activation of monocyte-macrophages by the chemokine CX3CL1 also lead to production of ROS and subsequent activation of TRPA1 (Old et al., 2014). Additionally, vincristine and paclitaxel enhanced the binding of the STAT3 (Signal Transducer and Activator of Transcription 3) to the CXCL12 gene promotor (Xu et al., 2017), causing up-regulation of C-X-C Motif Chemokine Ligand 12 in dorsal horn ganglia. CXCL12 is a member of the integrin family and acts as a ligand of the CXCR4 (CD184, C-X-C chemokine receptor type 4) and as an attractant for T-lymphocytes and monocytes. The activation of CXCR4 receptors in turn leads to an increase in intracellular Ca^2+^ and chemotaxis of immune cells to the site of inflammation (Chatterjee et al., 2014). Consequently, activation of the immune system, recruitment of immune cells and neuroinflammation should be considered as a putative mechanism contributing to the development of vincristine-induced neuropathy specifically, and CIPN more broadly.

## Prevention therapies and treatments

The development of CIPN is likely multifactorial and involves several mechanisms as discussed above, although it is currently unclear which effect(s) initiate the pathological cascade leading to neuronal dysfunction. Despite clear similarities in both the pathological mechanisms and clinical symptomatology of CIPN, the pathophysiology of CIPN nonetheless is thought to be compound-specific, with mechanism-specific prevention or treatment being the overarching clinical aim. In addition to providing targeted neuroprotection and relief from symptoms, any putative treatment for CIPN also must not interfere with the anti-tumor effects of the causative chemotherapeutic agent. Unfortunately, while the efficacy of a range of approaches preventing the development of CIPN including neuroprotectants and nutraceuticals (Table 1) have been evaluated in clinical trials, consistent beneficial effects have not as yet been shown for any single agent. Accordingly, current treatment strategies are predominantly based on modification of the chemotherapy regimen, including alteration of the dose, treatment cycles, timing, dosage form, and duration, as well as symptomatic management using a range of pharmacological approaches (Table 2). These include treatments targeting the neuropathic component in CIPN, including anticonvulsants (gabapentin, carbamazepine, oxcarbazepine, lamotrigine, topiramate) and antidepressants (amitriptyline, nortriptyline, venlafaxine, duloxetine). However, as is the case for many types of pain, these agents do not provide a satisfactory level of relief for many patients, with further research required to identify additional preventative or curative approaches.

**Table 1:** List of clinically used neutraceutical agents for prevention of the development of CIPN.

**Nutraceutical agent**	**Class**	**Chemotherapy agent**	**Study outcome**
**NUTRACEUTICAL AGENTS**			
Vitamin E	Vitamins	Cisplatin	+Decreased incidence and severity of peripheral neurotoxicity (Pace et al., 2003)
			+Reduced risk of developing neurotoxicity (Pace et al., 2010)
			+Neuroprotective effect (Argyriou et al., 2005)
		Oxaliplatin	−No significant decrease in the incidence of acute CIPN (de Afonseca et al., 2013)
		Paclitaxel	+Neuroprotective effect (Argyriou et al., 2005)
Glutamine	α-amino acid	Cisplatin	±Possible reduction of severity of CIPN symptoms (Huang et al., 2015)
		Vincristine	+Improvement in sensory function and self-reported overall quality of life (Sands et al., 2017).
		Oxaliplatin	±Possible reduction of severity of CIPN symptoms (Huang et al., 2015)
			+Reduction in incidence and severity of CIPN (Wang et al., 2007)
		Paclitaxel	±Possible reduction in the severity of CIPN (Vahdat et al., 2001)
			+Significant reduction of weakness, loss of vibratory sensation and toe numbness (Stubblefield et al., 2005)
Alpha-lipoic acid	Antioxidant	Cisplatin	−Ineffective at preventing neurotoxicity (Guo et al., 2014)
		Oxaliplatin	−Ineffective at preventing neurotoxicity (Guo et al., 2014)
			+Reduced severity of CIPN (Gedlicka et al., 2002)
Glutathione	Antioxidant	Cisplatin	+Prevention of neuropathy (Cascinu et al., 1995)
			±Possible neuroprotection (Colombo et al., 1995)
			±Possible decrease of severity of CIPN (Smyth et al., 1997)
		Oxaliplatin	×Increased resistance to platinum agents (Arrick and Nathan, 1984)
			+Possible prevention of CIPN (Cascinu et al., 2002)
Calcium/magnesium	Ions	Oxaliplatin	×Decreased antitumor efficacy of FOLFOX regimen in combination with Ca^2+^/Mg^2+^ infusions (Khattak, 2011; Wen et al., 2013)
			±Possible reduction in incidence and intensity of acute CIPN symptoms (Gamelin et al., 2004)
N-acetyl cysteine	Antidot	Oxaliplatin	±Possible reduction in incidence of CIPN (Lin et al., 2006)
Acetyl-L-carnitine	Amino acid	Taxanes	×Worsening of CIPN (Hershman et al., 2013)

*The outcome of each study is marked with +, positive effect on CIPN; −, no effect on CIPN; ±, potential positive effect on CIPN; ×, negative effect on CIPN or antitumor therapy*.

**Table 2:** Summary of clinical studies investigating the efficacy of pharmacological agents for symptomatical treatment of CIPN.

**Pharmacological agent**	**Class**	**Chemotherapy agent**	**Study outcome**
**PHARMACOLOGICAL AGENTS**			
Amifostine	Cyto-protective adjuvant	Cisplatin	+Reduction of neurotoxicity (Kemp et al., 1996; Planting et al., 1999; Hilpert et al., 2005)
			+Reduction in ototoxicity (Rubin et al., 1995; Fouladi et al., 2008)
			±Possible reduction of neurotoxicity (Lorusso et al., 2003)
		Oxaliplatin	+Decrease of severity of CIPN (Penz et al., 2001)
		Paclitaxel	−Ineffective in preventing or reducing neurotoxicity (Gelmon et al., 1999; Leong et al., 2003; Moore et al., 2003; Openshaw et al., 2004)
			+Reduction of neurotoxicity (Kanat et al., 2003; Lorusso et al., 2003; De Vos et al., 2005; Hilpert et al., 2005)
Carbamazepine Oxcarbazepine	Anticonvulsant	Oxaliplatin	+Prevention of CIPN (Eckel et al., 2002; Argyriou et al., 2006)
			−No prevention of CIPN (Wilson et al., 2002; von Delius et al., 2007)
Calcium channel blocker	−	Oxaliplatin	+Inhibits the development of acute peripheral neuropathy (Tatsushima et al., 2013)
Gabapentin	Anticonvulsant	Vinca alkaloids, platinum derivates, taxanes	−No prevention of CIPN (Rao et al., 2007)
Lamotrigine	Anticonvulsant	Paclitaxel, docetaxel, carboplatin, cisplatin, oxaliplatin, vincristine and vinblastine	−Not effective for relieving neuropathic symptoms (Rao et al., 2008)
Etanercept	Tumor necrosis factor (TNF) blocker	Cisplatin	±Transient analgesia and delay in development of cisplatin induced mechanical allodynia (Park H. J. et al., 2013; Vilholm et al., 2014)
Pregabalin	Anticonvulsant	Oxaliplatin	+Significant reduction of the severity of sensory neuropathy(Saif et al., 2010)
Amitriptyline	Tricyclic antidepressants	vinca alkaloids, platinum derivatives or taxanes	−No improvement of sensory neuropathic symptoms (Kautio et al., 2008)
Nortriptyline	Tricyclic antidepressants	Cisplatin	−No effect on paresthesia or pain (Hammack et al., 2002)
Venlafaxine	Selective serotonin and norepinephrine reuptake inhibitor (SNRI)	Paclitaxel	+Reduction of paresthesia (Durand and Goldwasser, 2002)
		Oxaliplatin	+Pain relief and a significant autonomy improvement (Durand et al., 2005)
			+Paresthesia improvement(Durand et al., 2003)
Duloxetine	Selective serotonin and norepinephrine reuptake inhibitor (SNRI)	Vinca alkaloids, platinum derivatives or taxanes	+Reduction in pain (Smith et al., 2013)
Topiramate	Anticonvulsant	Oxaliplatin	+Pain relief and a significant autonomy improvement (Durand et al., 2005)

## Summary and conclusions

CIPN is a common side effect of cancer chemotherapy that adversely affects the quality of life of patients. Although the individual biological effects of these chemicals on cancer cells are relatively well known and studied extensively, the precise mode of action on peripheral nerves is not always clear. The development of CIPN is likely multifactorial and involves effects on neuronal and/or mitochondrial DNA and gene expression, axonal transport, ion channel expression and function, as well as neuroimmune mechanisms. While rodent models—particularly those avoiding confounding physiological effects of high systemic chemotherapy doses—have provided valuable insight, validation of the mechanisms of CIPN in patients has been difficult not least because of the extensive co-morbidities usually present in this patient group. Nonetheless, significant advances in our understanding of the pathophysiological mechanisms of CIPN have been made, which will hopefully lead to improved clinical management of this devastating side effect in future.

## Author contributions

All authors listed, have made substantial, direct and intellectual contribution to the work, and approved it for publication.

### Conflict of interest statement

The authors declare that the research was conducted in the absence of any commercial or financial relationships that could be construed as a potential conflict of interest.
